# Hyaluronidase-1 mediates postprandial suppression of hepatic gluconeogenesis

**DOI:** 10.1093/lifemeta/loaf016

**Published:** 2025-05-13

**Authors:** Xi Chen, Sophie Dogné, Yanru Deng, Huiqiao Li, Jieyi Meng, Charlise Giang, Jan-Bernd Funcke, Leon G Straub, Michelle Dias, Sundararajah Thevananther, Qiang Tong, Abu Hena Mostafa Kamal, Chandra Shekar R Ambati, Yu’e Liu, Nagireddy Putluri, Xia Gao, Miao-Hsueh Chen, Dongyin Guan, Hari Krishna Yalamanchili, Shangang Zhao, Nathalie Caron, Yi Zhu

**Affiliations:** USDA/ARS Children’s Nutrition Research Center, Department of Pediatrics, Baylor College of Medicine, Houston, TX 77030, United States; Molecular Physiology Research Unit (URPhyM), Namur Research Institute for Life Sciences (NARILIS), University of Namur, Namur 5000, Belgium; USDA/ARS Children’s Nutrition Research Center, Department of Pediatrics, Baylor College of Medicine, Houston, TX 77030, United States; USDA/ARS Children’s Nutrition Research Center, Department of Pediatrics, Baylor College of Medicine, Houston, TX 77030, United States; USDA/ARS Children’s Nutrition Research Center, Department of Pediatrics, Baylor College of Medicine, Houston, TX 77030, United States; USDA/ARS Children’s Nutrition Research Center, Department of Pediatrics, Baylor College of Medicine, Houston, TX 77030, United States; Touchstone Diabetes Center, University of Texas Southwestern Medical Center at Dallas, Dallas, TX 75390, United States; Department of Biochemistry and Molecular Cell Biology, University Medical Center Hamburg-Eppendorf, Martinistr. 52, 20246 Hamburg, Germany; USDA/ARS Children’s Nutrition Research Center, Department of Pediatrics, Baylor College of Medicine, Houston, TX 77030, United States; Jan and Dan Duncan Neurological Research Institute at Texas Children’s Hospital, Houston, TX 77030, United States; Division of Gastroenterology, Department of Pediatrics, Baylor College of Medicine, Houston, TX 77030, United States; USDA/ARS Children’s Nutrition Research Center, Department of Pediatrics, Baylor College of Medicine, Houston, TX 77030, United States; Department of Molecular and Cellular Biology, Baylor College of Medicine, 1 Baylor Plaza, Houston, TX 77030, United States; Advanced Technology Cores, Dan L Duncan Comprehensive Cancer Center, Baylor College of Medicine, 1 Baylor Plaza, Houston, TX 77030, United States; Department of Pediatric Hematology-Oncology, Boston Children’s Hospital, Dana Farber Cancer Institute, Harvard Medical School, Boston, MA 02115, United States; Department of Molecular and Cellular Biology, Baylor College of Medicine, 1 Baylor Plaza, Houston, TX 77030, United States; USDA/ARS Children’s Nutrition Research Center, Department of Pediatrics, Baylor College of Medicine, Houston, TX 77030, United States; USDA/ARS Children’s Nutrition Research Center, Department of Pediatrics, Baylor College of Medicine, Houston, TX 77030, United States; Endocrinology, Department of Medicine, Baylor College of Medicine, Houston, TX 77030, United States; USDA/ARS Children’s Nutrition Research Center, Department of Pediatrics, Baylor College of Medicine, Houston, TX 77030, United States; Jan and Dan Duncan Neurological Research Institute at Texas Children’s Hospital, Houston, TX 77030, United States; Barshop Institute for Longevity and Aging Studies, Division of Endocrinology, Department of Medicine, University of Texas Health Science Center at San Antonio, San Antonio, TX 78229, United States; Molecular Physiology Research Unit (URPhyM), Namur Research Institute for Life Sciences (NARILIS), University of Namur, Namur 5000, Belgium; USDA/ARS Children’s Nutrition Research Center, Department of Pediatrics, Baylor College of Medicine, Houston, TX 77030, United States

**Keywords:** HYAL1, hyaluronan, hepatic gluconeogenesis, metabolites, mitochondrial function

## Abstract

Hepatic gluconeogenesis is a critical process that generates glucose from non-carbohydrate precursors during fasting to support vital organs like the brain and red blood cells. Postprandially, this process is rapidly suppressed to allow for glucose storage as glycogen and lipids in the liver. Failure to suppress gluconeogenesis after meals leads to elevated postprandial glucose levels, a key feature of type 2 diabetes. This dynamic switch is regulated by insulin and glucagon, but insulin resistance impairs this regulation. In this study, we identified a novel mechanism involving postprandial circulating hyaluronan (HA) and lysosomal hyaluronidase-1 (HYAL1) that suppresses hepatic gluconeogenesis by rewiring hepatic metabolism and mitochondrial function. *Hyal1* knockout (*Hyal1* KO) mice exhibited increased gluconeogenesis, while liver-specific *Hyal1* overexpression (Liv-*Hyal1*) mice showed reduced gluconeogenic activity. Transcriptomic analysis revealed minimal changes in liver gene expression due to *Hyal1* deletion, but metabolomic profiling demonstrated that *Hyal1* overexpression mitigated high-fat diet (HFD)-induced elevations in gluconeogenic pathway metabolites. Mechanistically, HYAL1-mediated HA digestion activates a feedback loop in HA synthesis, repartitioning the cellular uridine diphospho-N-acetyl-D-glucosamine (UDP-GlcNAc) pool. This reduces O-linked N-acetylglucosamine modification (O-GlcNAcylation) of mitochondrial ATP synthase subunits, decreasing ATP production and suppressing gluconeogenesis. Importantly, this pathway remains intact in the livers of HFD-fed, insulin-resistant mice. In summary, our findings reveal a new postprandial mechanism for regulating hepatic gluconeogenesis, highlighting the potential of enhancing postprandial HA levels or hepatic HYAL1 activity as a therapeutic strategy for managing excessive gluconeogenesis in insulin-resistant conditions, such as type 2 diabetes.

## Introduction

Hyaluronic acid (abbreviated HA), or hyaluronan, is a linear glycosaminoglycan with simple repeating disaccharide units of D-glucuronic acid (GlcA or GlcUA) and N-acetyl-D-glucosamine (GlcNAc) [1, 2]. HA is a significant extracellular matrix component with many critical physiological functions [3]. It is synthesized from UDP-GlcA and UDP-GlcNAc at the plasma membrane by several HA synthases, and the nascent HA chain is extruded through the plasma membrane into the extracellular space [4]. Deletion of the predominant embryonic HA synthase isoform *Has2* in mice leads to the lethality of the embryos at mid-gestation (E9.5–10) [5]. Extracellular HA is mostly degraded locally by a family of hyaluronidases (HYALs), and fragmented HA is extracted by lymph and eventually enters the bloodstream [6]. HA is abundant in circulation in a molecular weight range of 100–300 kDa [7], but with an extremely short half-life of 2–3 min, suggesting a rapid turnover in circulation [8]. The liver is the primary organ for clearing circulating HA [9, 10].

Hyaluronidase-1 (HYAL1) and HYAL2 are the two most ubiquitously expressed HYALs. They work together to degrade HA in a stepwise fashion [6], with HYAL2 degrading HA at the cell surface into around 20 kDa fragments, which are then engulfed and delivered to strongly acidic lysosomes for final degradation by HYAL1 and other exoglucosidases [11]. HYAL2 deficiency induces a buildup of very high molecular weight (MW) HA (> 3.10^6^ Da) in the lymph and serum, with severe lymph node distortion [12]. By contrast, HYAL1 deficiency leads to HA overload in the liver and a moderate increase in serum HA concentration without changes to HA MW distribution [12].

HA synthesis and degradation are closely integrated with glucose metabolism in the cell [13]. Synthesis of HA is an energy-consuming process. The major HA synthase, HAS2, is regulated by sirtuin 1 (SIRT1) and the AMP-activated protein kinase (AMPK), two critical energy sensors in the cell, allowing HA synthesis to be synchronized with cellular energy supply states. HA synthesis also depends critically on the size of the cytoplasmic UDP-sugar pool [13]. Glucose metabolism provides energy and precursors through glycolysis for HA biosynthesis; thus, glucose utilization pathways are tightly integrated with HA synthesis. On the other hand, the coordinated action of HYALs, β-glucuronidase, and hexosaminidase ensures the complete degradation of HA, providing GlcA and GlcNAc to the cell. GlcNAc is converted in GlcNAc-6-phosphate (GlcNAc-6-P) by the GlcNAc kinase in the GlcNAc salvage pathway [14, 15] to sustain the synthesis of UDP-GlcNAc for complex glycoconjugates. Vertebrates do not recycle GlcA to GlcA-1-phosphate and then to UDP-GlcA; instead, they convert GlcA into xylulose-5-phosphate through several redox and decarboxylation reactions [13].

After a meal, circulating HA surges in parallel with the shutting down of gluconeogenesis. However, whether the postprandial HA surge suppresses gluconeogenesis has not been investigated. We showed that the systemic deletion of *Hyal1* impaired glucose tolerance in mice treated with HFD, partly due to elevated gluconeogenesis. Liver-specific *Hyal1*-overexpression mice showed improved glucose tolerance and suppressed gluconeogenesis from pyruvate during the HFD challenge. Measurements of metabolites, the mitochondrial protein O-linked N-acetylglucosamine modification (O-GlcNacylation), and cellular energetics showed an unexpected regulation of hepatocyte gluconeogenesis by HYAL1 via O-GlcNacylation of the mitochondrial ATP synthase subunits. This work links intracellular HA digestion to the regulation of gluconeogenesis in hepatocytes.

## Results

### Postprandial increase in circulating HA levels

**Figure 1:**
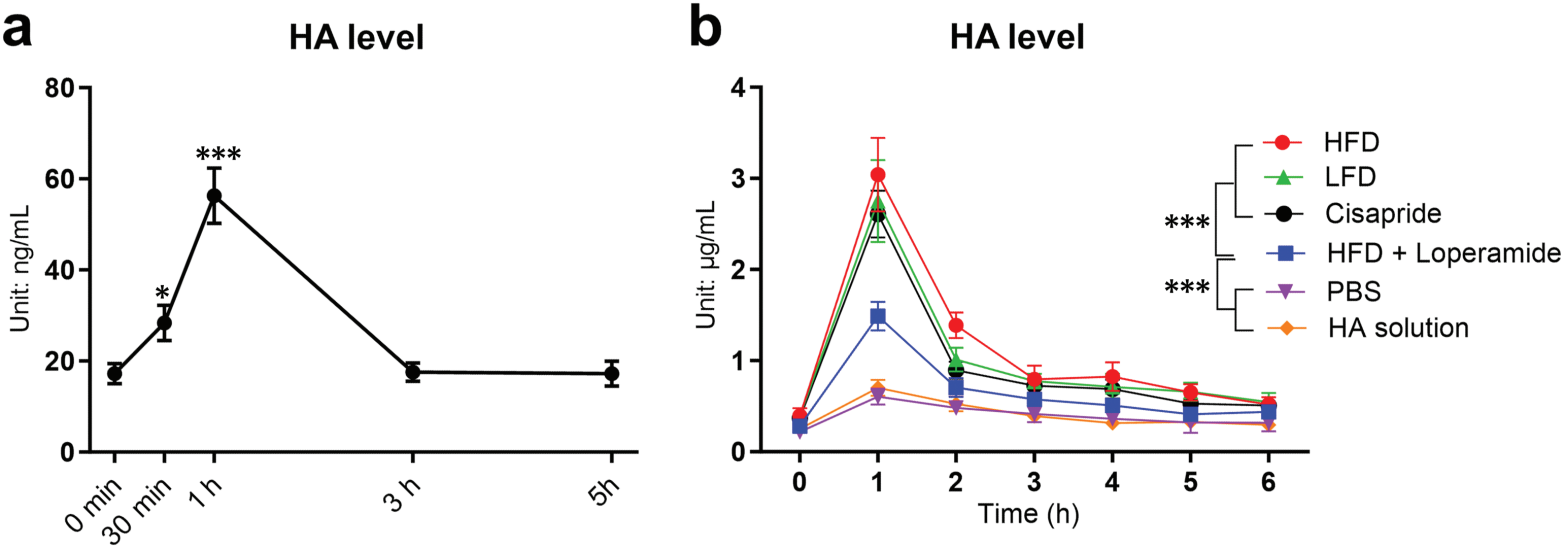
Postprandial increase of circulating HA levels. (a) HA levels after a mixed meal in human subjects. The plot is a reanalysis of our previously published data [16]. *n* = 30. (b) HA levels in WT mice subjected to LFD, HFD, oral garage with PBS, HA solution (5 mg/kg body weight), cisapride (0.5 mg/kg body weight via oral gavage), or a combination of HFD and loperamide (1.2 mg/kg body weight via oral gavage), *n* = 6 mice per group. Serial *t*-tests were used to compare different time points with time = 0 min for panel (a), and two-way ANOVA followed by *post-hoc* Sidak multiple comparisons at different time points for panel (b). All results are shown as mean ± SEM.^*^P < 0.05; ^***^P < 0.001.

There is a postprandial surge of serum HA levels in human subjects that peaks 1 h after a meal (Fig. 1a). This surge can also be observed in mice (Fig. 1b). Specifically, solid food, including low-fat diet (LFD) and HFD, elicits a quick surge in serum HA levels, whereas PBS and even HA solution fail to elicit such a surge. We reason that this is because HA cannot be absorbed through the intestinal tract, and the increase in serum HA results from intestinal movement and the release of HA from the tissue interstitial space. To test that, we used cisapride, a drug that increases motility in the upper gastrointestinal tract, and loperamide, a drug that inhibits the movement of the gastrointestinal tract. Cisapride treatment, even without solid food, was sufficient to elicit an HA surge similar to LFD or HFD, and treatment of mice with loperamide during feeding with HFD significantly blunted the increase of HA in the circulation (Fig. 1b). Gut-released HA is carried via the inferior vena cava to the portal vein and filtered out through the liver. HYAL1 and HYAL2 are two major enzymes that degrade HA in the liver. Gene expression levels of the two enzymes were not significantly increased after a meal (Supplementary Fig. S1a). Interestingly, the expression levels of HA synthases, especially *Has3*, were significantly increased 1 h after refeeding (Supplementary Fig. S1b). Given that HYAL1 is the specific enzyme responsible for intracellular HA degradation, we decided to investigate *Hyal1* in postprandial HA digestion and metabolic changes.

### *Hyal1* deficiency increases gluconeogenesis from pyruvate

Systemic deletion of *Hyal1* resulted in a significant increase in serum HA levels on both LFD and HFD (Supplementary Fig. S2a). However, there was no further increase of serum HA in *Hyal1* KO mice on HFD compared with that on LFD (Supplementary Fig. S2a). Gene expression analysis showed *Hyal1* was completely absent from the liver. Deletion of *Hyal1* did not lead to a compensatory increase of *Hyal2* or *Hyal3* in the liver (Supplementary Fig. S2b). *Hyal1* was also the only HYAL isoform that upregulated by HFD in the liver (Supplementary Fig. S2b). Deletion of *Hyal1* led to a reduction in the expression of *Has2* and *Has3*, the two most abundant HA synthase isoforms in the liver (Supplementary Fig. S2c). In the adipose tissue, HFD increased the expression of *Hyal1*, *Hyal2*, and *Hyal3*. *Hyal1* deletion had no effect on *Hyal2* or *Hyal3* expression (Supplementary Fig. S2d). Similar to what was observed in the liver, *Hyal1* deletion led to a reduction in *Has1* and *Has3* expression, mostly on LFD (Supplementary Fig. S2e), suggesting a common feedback mechanism to reduce HA synthesis after the blocking of HA degradation by *Hyal1* deletion.

**Figure 2:**
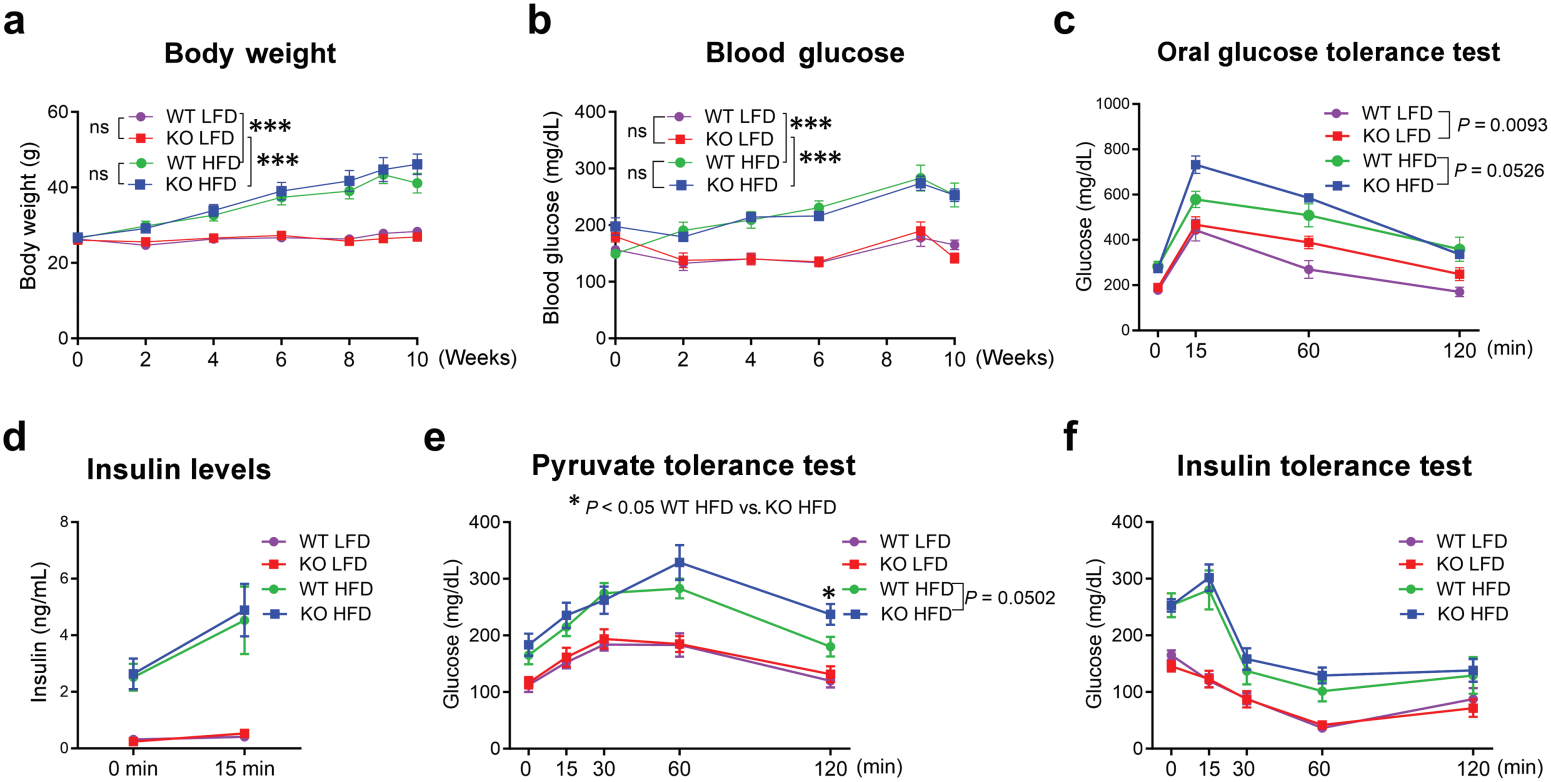
*Hyal1* deficiency impairs glucose homeostasis. (a) Body weight. (b) Glycemia in random fed state. (c) Oral glucose tolerance test (OGTT). (d) Serum insulin levels during OGTT. (e) PTT. (f) ITT of male WT and *Hyal1* KO mice on LFD and HFD for 8−10 weeks. In panels (a)–(f), *n* = 6 WT mice on LFD; *n* = 8 WT mice on HFD; *n* = 7 *Hyal1* KO mice on LFD or HFD. Two-way ANOVA followed by *post-hoc* Sidak multiple comparison test was used at different time points between WT and *Hyal1* KO mice on LFD or HFD. All results are shown as mean ± SEM. *P* values or asterisks indicate statistical differences in *y* values at indicated time points between WT and *Hyal1* KO mice on HFD. ns, not significant. ^*^P < 0.05; ^**^P < 0.01; ^***^P < 0.001.

To understand the role of *Hyal1* in the development of metabolic dysfunction, we placed a cohort of male *Hyal1* KO mice and their littermate control WT mice on LFD or HFD for 11 weeks. WT mice gained weight (Fig. 2a) and developed hyperglycemia (Fig. 2b) on HFD as compared to LFD. Deletion of *Hyal1* did not affect body weight (Fig. 2a) or hyperglycemia (Fig. 2b) relative to WT mice on either LFD or HFD. *Hyal1* KO mice on HFD showed higher serum glucose levels at the early timepoints after glucose challenge (Fig. 2c), with similar hyperglycemia-induced insulin levels (Fig. 2d). *Hyal1* KO mice fed HFD also showed higher glucose levels during a pyruvate tolerance test (PTT) after overnight fasting, indicating higher gluconeogenesis from pyruvate (Fig. 2e). Sensitivity to intraperitoneally injected insulin did not differ between *Hyal1* KO and WT mice (Fig. 2f). By contrast, deletion of *Hyal1* did not affect serum lipids (total cholesterol, direct high-density lipoprotein, triglycerides, and non-esterified fatty acids (NEFA)) (Supplementary Fig. S2f). In female mice, *Hyal1* KO mice showed no difference in body weight development (Supplementary Fig. S3a) or glycemia (Supplementary Fig. S3b) in comparison to littermate WT mice on LFD. *Hyal1* KO females already developed mild glucose intolerance on LFD (Supplementary Fig. S3c) with no difference in glucose-stimulated insulin levels (Supplementary Fig. S3d), which was probably due to increased gluconeogenesis (Supplementary Fig. S3e). Insulin sensitivity was not changed; a ticking up of glucose at 120 min in the *Hyal1* KO group might be another indication of higher gluconeogenesis while recovering from hypoglycemia induced by insulin treatment (Supplementary Fig. S3f).

### Hepatic *Hyal1* overexpression reduces gluconeogenesis in mice on HFD

**Figure 3:**
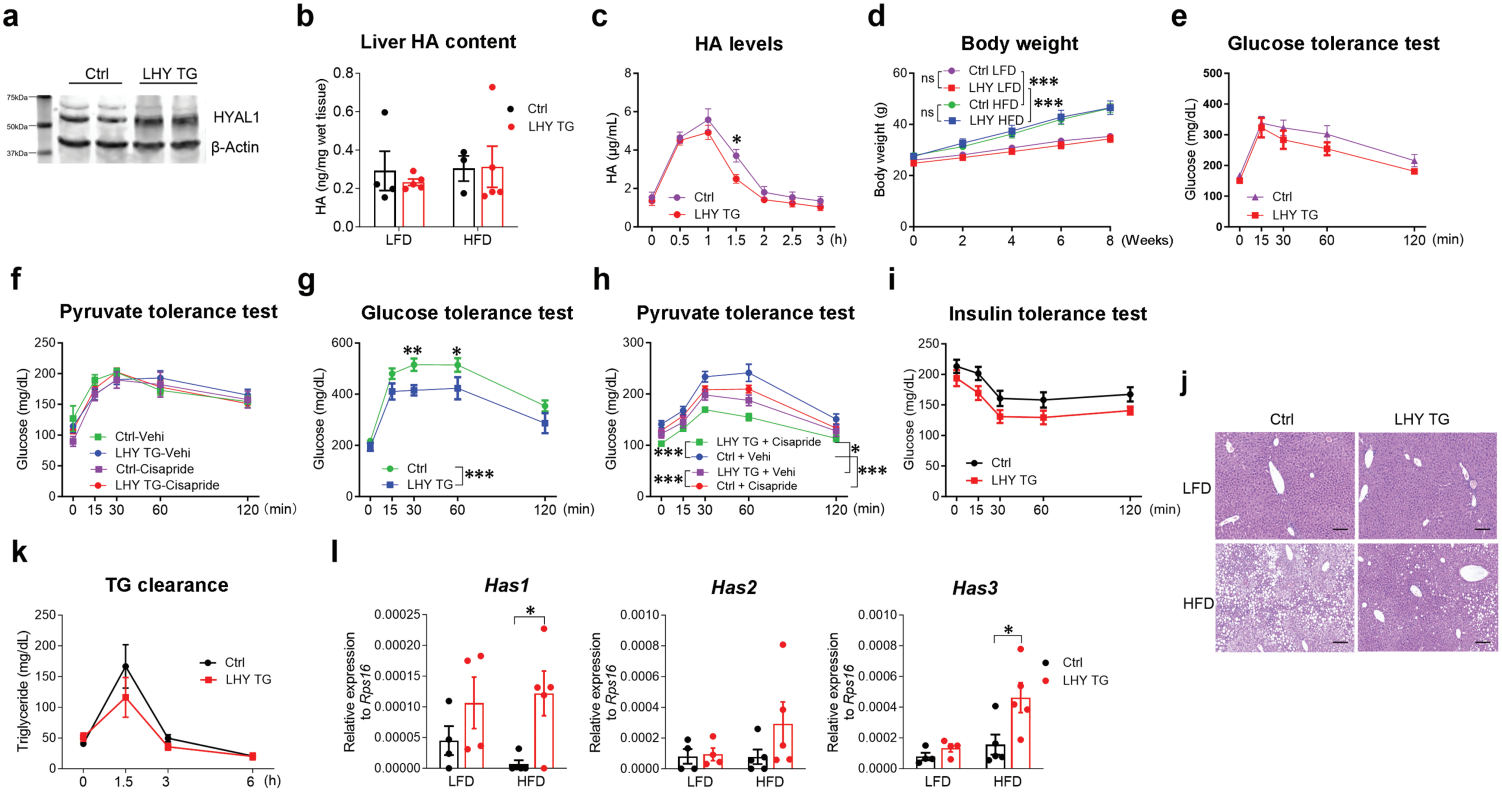
Hepatic *Hyal1* overexpression improves glucose metabolism. (a) HYAL1 protein levels in the livers of control and LHY TG mice after 1-week Dox200 LFD treatment. (b) Hepatic HA content of control and LHY TG mice treated with Dox200 LFD or HFD for 8 weeks. *n* = 4 and 5 for control and LHY TG mice, respectively, on LFD; *n* = 3 and 5 for control and LHY TG mice, respectively, on HFD. (c) Postprandial serum HA levels in control and LHY TG mice after overnight fasting. *n* = 4 for control, 5 for LHY TG group. (d) Body weight of male control and LHY TG mice treated with Dox200 LFD or Dox200 HFD for 8 weeks. *n* = 11 and 5 for control and LHY TG mice, respectively, on LFD; *n* = 11 and 10 for control and LHY TG mice, respectively, on HFD. (e) GTT of control and LHY TG mice treated with Dox200 LFD for 8 weeks. *n* = 11 and 5 control and LHY TG mice, respectively. (f) PTT of control and LHY TG mice treated with Dox200 LFD for 9 weeks, with or without cisapride pretreatment (0.5 mg/kg body weight, oral gavage). *n* = 4, 4, 5, and 5 mice for control + vehicle, control + cisapride, LHY TG + vehicle, and LHY TG + cisapride groups, respectively. (g) GTT of control and LHY TG mice treated with Dox200 HFD for 8 weeks. *n* = 16 and 9 for control and LHY TG mice, respectively. (h) PTT of control and LHY TG mice treated with Dox200 HFD for 9 weeks, with or without cisapride pretreatment (0.5 mg/kg body weight, oral gavage). *n* = 16, 7, 9, and 9 mice for control + vehicle, control + cisapride, LHY TG + vehicle, and LHY TG + cisapride groups, respectively. (i) ITT of control and LHY TG mice treated with Dox200 HFD for 11 weeks; *n* = 16 and 9 mice for control and LHY TG groups, respectively. (j) Representative H&E staining images of control and LHY TG mice treated with Dox200 LFD or Dox200 HFD for 12 weeks. Scale bar = 200 µm. (k) Clearance of orally gavaged intralipid in the circulation by control and LHY TG mice treated with Dox200 HFD for 10 weeks. *n* = 10 and 6 mice for control and LHY TG groups, respectively. (l) Hepatic expression of HA synthase genes in control and LHY TG mice treated with Dox200 HFD for 8 weeks. *n* = 4 control and LHY TG mice on Dox200 LFD; *n* = 5 control and LHY TG mice on Dox200 HFD. Two-way ANOVA followed by *post-hoc* Sidak multiple comparison test at different time points for control and LHY TG mice on LFD or HFD for panels (c)−(k); asterisks indicate statistical differences in *y* values at an indicated time point between control and LHY TG mice on HFD for panel (g). Two-way ANOVA tests were used for panels (b) and (l), and additional *post-hoc* analysis was performed for panel (l). All results are shown as mean ± SEM. ns, not significant. ^*^P < 0.05; ^**^P < 0.01; ^***^P < 0.001.

As the liver is the major gluconeogenic organ [17], we focused on it next. Hepatic *Hyal1* expression increased quickly after mice were treated with HFD (Supplementary Fig. S4a). To determine whether this was an adaptation to HFD, we generated a doxycycline-inducible liver-specific *Hyal1*-overexpression mouse Alb-Cre/Rosa-rtTA/TRE-*Hyal1*, referred to as LHY TG hereafter. It showed a > 100-fold overexpression of the *Hyal1* gene in the liver, no leaky expression in the heart (Supplementary Fig. S4b), and a moderate increase in the HYAL1 protein (Fig. 3a). Hepatic overexpression of *Hyal1* did not result in a reduction in hepatic HA content (Fig. 3b) or serum HA levels (Supplementary Fig. S4c); however, they increased postprandial clearance of HA, as they showed a similar spike to control mice but a faster reduction in serum HA levels after a meal (Fig. 3c). LHY TG mice gained similar body weight (Fig. 3d) and showed no difference in glucose tolerance test (GTT) (Fig. 3e) or PTT (Fig. 3f) compared to control mice (Alb-cre/Rosa-rtTA) on Dox200 LFD. However, when mice were treated with Dox200 HFD, despite similar weight gain (Fig. 3d), the LHY TG mice showed improved GTT (Fig. 3g) and a significant reduction in gluconeogenesis from pyruvate (Fig. 3h), independent of insulin sensitivity (Fig. 3i). In addition, when mice were treated with cisapride to increase gut mobility and circulating HA, the PTT levels in both the control and the LHY TG mice decreased significantly on HFD but not on LFD (Fig. 3f and h). HFD led to severe hepatic steatosis, and *Hyal1* overexpression significantly reduced it (Fig. 3j). However, there were no differences in how the mice handled exogenously administered lipids (Fig. 3k). Serum lipid species, including total cholesterol, high-density lipoprotein (HDL) cholesterol, triglycerides, and NEFA, were not affected by *Hyal1* overexpression in mice on LFD or HFD (Supplementary Fig. S4d). Interestingly, *Hyal1* overexpression markedly upregulated hepatic *Has* expression in mice on HFD (Fig. 3l). Together with the downregulation of *Has* expression observed following *Hyal1* deletion in the liver (Supplementary Fig. S2c), these data suggest a reciprocal regulation of HA synthesis by HA degradation in hepatocytes.

### The effect of *Hyal1* on gluconeogenesis is transcription independent

**Figure 4:**
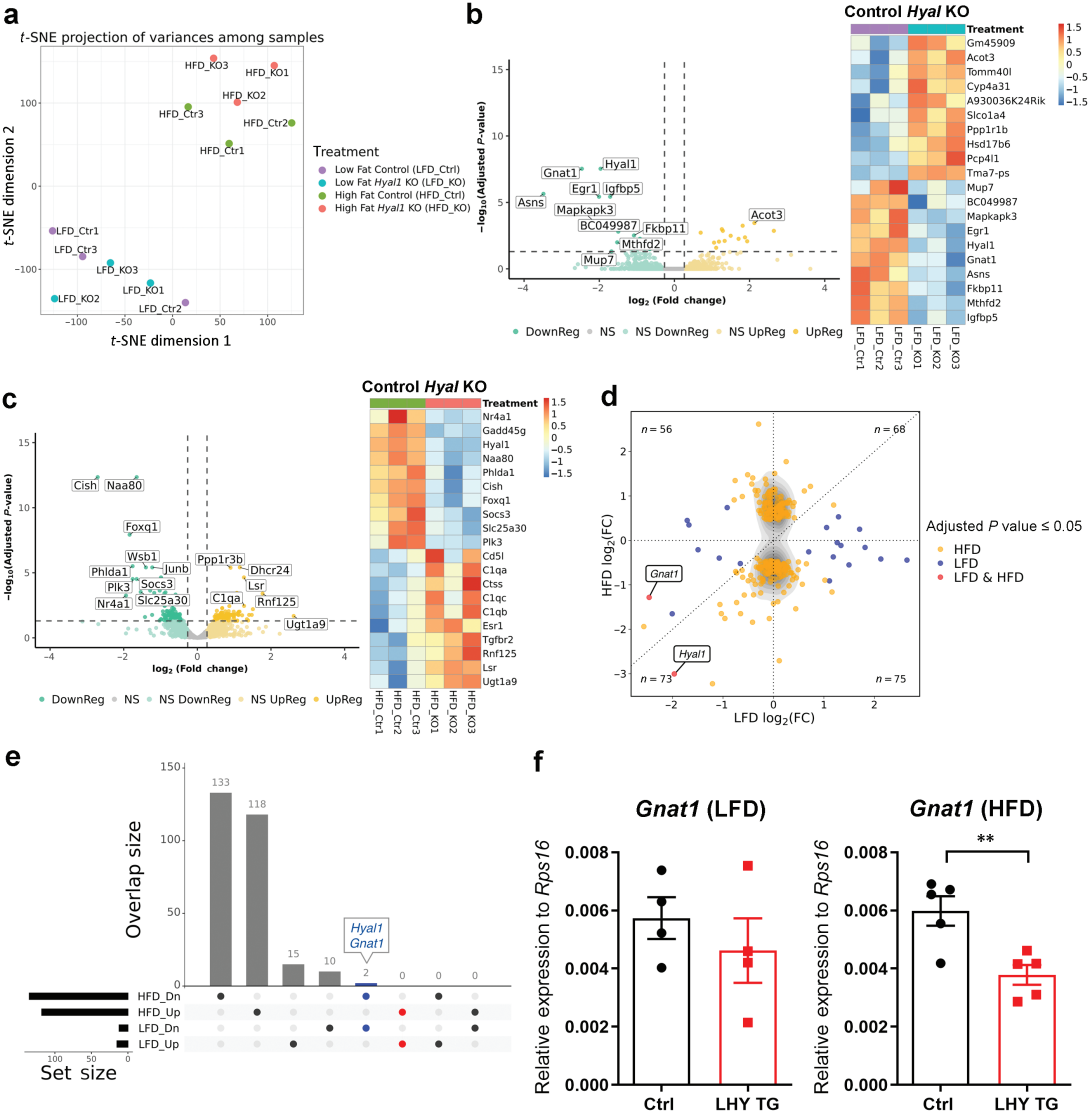
*Hyal1* knockout does not significantly affect gene expression in the liver. (a) *t*-SNE projection of variances among samples using perplexities of 1 and 1,000 iterations. (b) Volcano plot of genes significantly upregulated and downregulated in *Hyal1* KO livers on LFD, with a horizontal line indicating adjusted *P* value cutoff (≤ 0.05) and vertical lines indicating log_2_(Fold change) cutoffs (≤ − 0.263 or ≥ 0.263). A heat map of the ten most upregulated and ten most downregulated genes by log_2_(Fold change) is shown on the right. (c) Volcano plot of genes significantly upregulated and downregulated upon *Hyal1* KO on HFD, with a horizontal line indicating adjusted *P* value cutoff (≤ 0.05) and vertical lines indicating log_2_(Fold change) cutoffs (≤ −0.263 or ≥ 0.263). A heat map of the ten most upregulated and ten most downregulated genes by log_2_(Fold change) is shown on the right. (d) Correlation of significantly changed genes (adjusted *P* value ≤ 0.05) by *Hyal1* deletion in the liver on LFD and HFD. (e) Only two genes were regulated by *Hyal1* deletion in the same direction on LFD and HFD treatments. LFD_Dn: genes downregulated by *Hyal1* deletion in the liver for mice on LFD. LFD_Up: genes upregulated by *Hyal1* deletion in the liver for mice on LFD. HFD_Dn: genes downregulated by *Hyal1* deletion in the liver for mice on HFD. HFD_Up: genes upregulated by *Hyal1* deletion in the liver for mice on HFD. (f) *Gnat1* expression in the liver from control and liver *Hyal1*-overexpressing (LHY TG) mice kept on LFD (left) or HFD (right) for 12 weeks. *n* = 4 mice on LFD; *n* = 5 mice on HFD. Results are shown as mean ± SEM. ^**^P < 0.01, unpaired two-tailed *t*-test.

To understand how *Hyal1* might regulate gluconeogenesis in the liver, we performed an RNA sequencing (RNA-seq) of WT and *Hyal1* KO livers harvested from mice kept on LFD and HFD. The *t*-distributed stochastic neighbor embedding (*t*-SNE) projection of variances among the samples revealed a major transcriptional difference determined by diet but not by genotype (Fig. 4a). Volcano plots comparing *Hyal1* KO to WT mice on LFD showed that few genes were significantly changed (Fig. 4b), and a similar lack of significantly altered genes between *Hyal1* KO and WT mice was evident for mice treated with HFD (Fig. 4c). Correlation of the significantly changed genes in the *Hyal1* KO mice with WT controls on LFD and HFD suggested that few genes showed the same direction of regulation (Fig. 4d). Only two genes showed the same direction of change (downregulation) with LFD and HFD, including *Hyal1* itself (Fig. 4e); the other was guanine nucleotide binding protein, alpha transducing activity polypeptide 1 (*Gnat1*). However, GNAT1 protein levels and reported functions are restricted to the retina [18, 19]. In addition, *Gnat1* expression was suppressed in *Hyal1*-overexpression liver tissue on HFD (Fig. 4f), similar to the results of *Hyal1* deletion in the liver, arguing against a possible role of *Gnat1* in the *Hyal1*-mediated metabolic phenotype. A complete list of the detected genes is in Supplementary Table S1.

### Hepatic *Hyal1* overexpression reduces metabolites in the gluconeogenesis pathway

**Figure 5:**
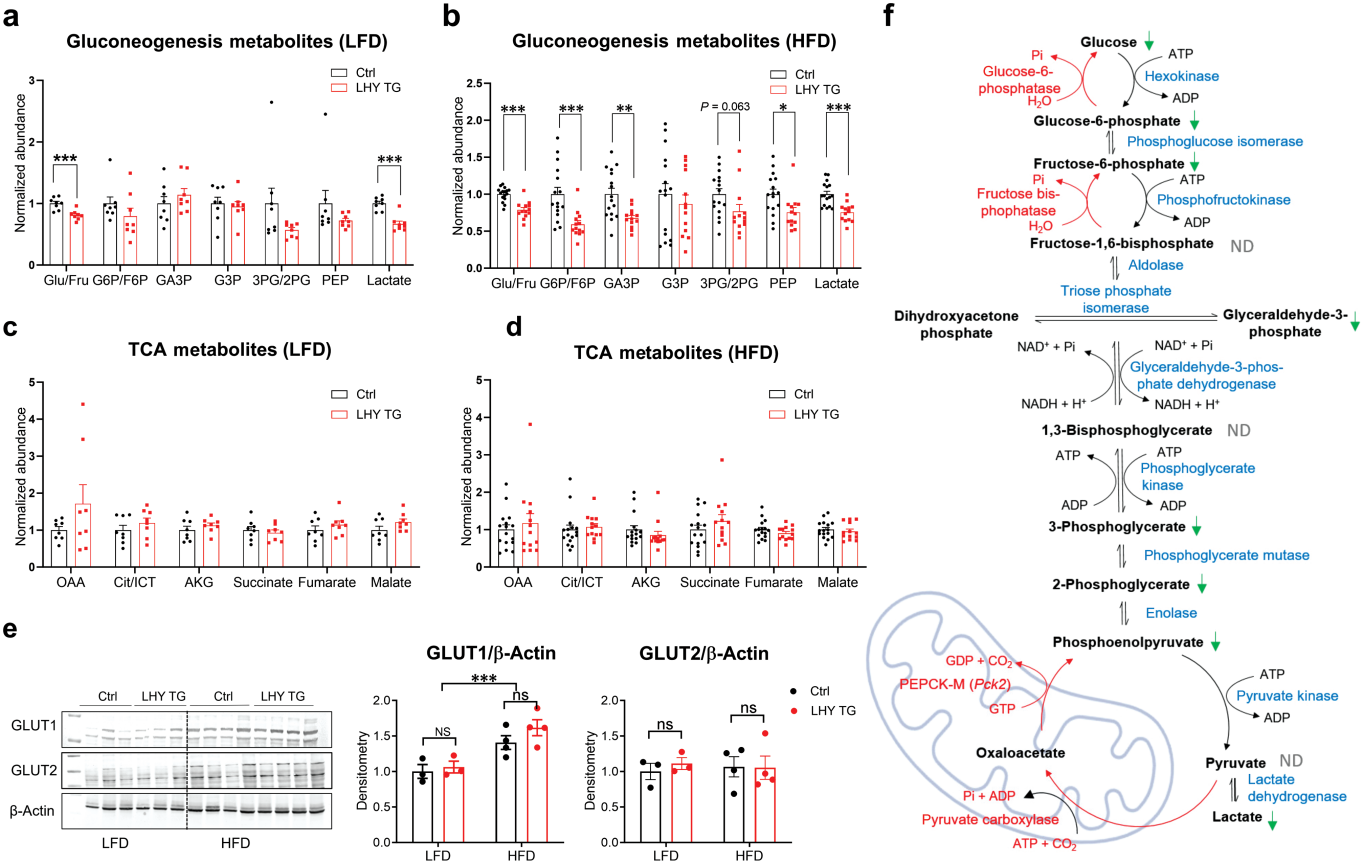
Hepatic *Hyal1* overexpression reduces metabolites in the gluconeogenesis pathway. (a) Hepatic metabolites from gluconeogenesis pathway in control and LHY TG mice kept on LFD. (b) Hepatic metabolites from gluconeogenesis pathway in control and LHY TG mice kept on 12-week HFD. (c) Hepatic metabolites from the TCA cycle pathway in control and LHY TG mice kept on LFD. (d) Hepatic metabolites from TCA cycle pathway in control and LHY TG mice kept on 12-week HFD. In panels (a) and (c), *n* = 8 mice for each group. In panels (b) and (d), *n* = 16 mice for the control group, *n* = 13 mice for the LHY TG group. Abbreviations of metabolites in panels (a)–(d): Glu: glucose; Fru: fructose; G6P: glucose-6-phosphate; F6P: fructose-6-phosphate; GA3P: glyceraldehyde-3-phosphate; G3P: glycerol-3-phosphate; 3PG/2PG: 3-phosphoglycerate/2-phosphoglycerate; PEP: phosphoenolpyruvate; OAA: oxaloacetic acid; Cit/ICT: citrate/isocitrate; AKG: α-keto glutarate. (e) Western blot analysis of GLUT1 and GLUT2 in liver lysates of control or LHY TG mice on LFD or HFD. Densitometry quantifications are shown on the right. Two-way ANOVA followed by *post-hoc* Sidak multiple comparison tests were performed. *n* = 3 and 4 mice for LFD and HFD groups, respectively. (f) Illustrations of metabolites changed in *Hyal1*-overexpressing liver from HFD mice in gluconeogenesis pathway. The green arrow indicates a statistically significant reduction in abundance in LHY TG liver lysates in comparison to control liver lysates. ND: not determined. All results are shown as mean ± SEM. ns, not significant. ^*^P < 0.05; ^**^P < 0.01; ^***^P < 0.001.

Because HYAL1 was unlikely to regulate gluconeogenesis by modulating gene expression, we decided to determine whether it directly affected metabolites in the liver. We measured metabolites from gluconeogenesis and the tricarboxylic acid (TCA) cycle pathway in control and *Hyal1*-overexpressing livers harvested from age-matched male mice treated with LFD or 12-week HFD. All mice were fasted overnight to allow their livers to enter a gluconeogenesis state. HFD significantly increased the pool of glucose/fructose, glucose-6-phosphate (G6P)/fructose-6-phosphate (F6P), and lactate (Supplementary Fig. S5a). The exact identities of glucose and fructose, as well as that of G6P and F6P, could not be distinguished by mass spectrometry. However, as glucose is much more abundant than fructose intracellularly, these data primarily reflected higher glucose concentrations in HFD livers. On LFD, *Hyal1* overexpression did not affect metabolites in the gluconeogenesis pathway much except for a mild reduction in glucose (Fig. 5a). On HFD, however, *Hyal1*-overexpressing liver had most of the gluconeogenesis pathway metabolites reduced (Fig. 5b). HFD feeding increased metabolites in the TCA cycle (Supplementary Fig. S5b), but *Hyal1* overexpression did not affect TCA metabolites on LFD and HFD (Fig. 5c and d). A complete list of relative abundances of metabolites measured is given in Supplementary Table S2. Plasma membrane glucose transporters GLUT1 and GLUT2 were not affected by *Hyal1* overexpression in the liver (Fig. 5e), excluding the possibility of glucose export as the cause of lower glucose levels in PTT in LHY TG mice. All these data indicate that HYAL1 directly modulates the intracellular metabolite pool in the gluconeogenesis pathway, mostly on HFD (Fig. 5f).

### HYAL1-mediated HA degradation reduces mitochondrial ATP production and inhibits gluconeogenesis in hepatocytes

**Figure 6:**
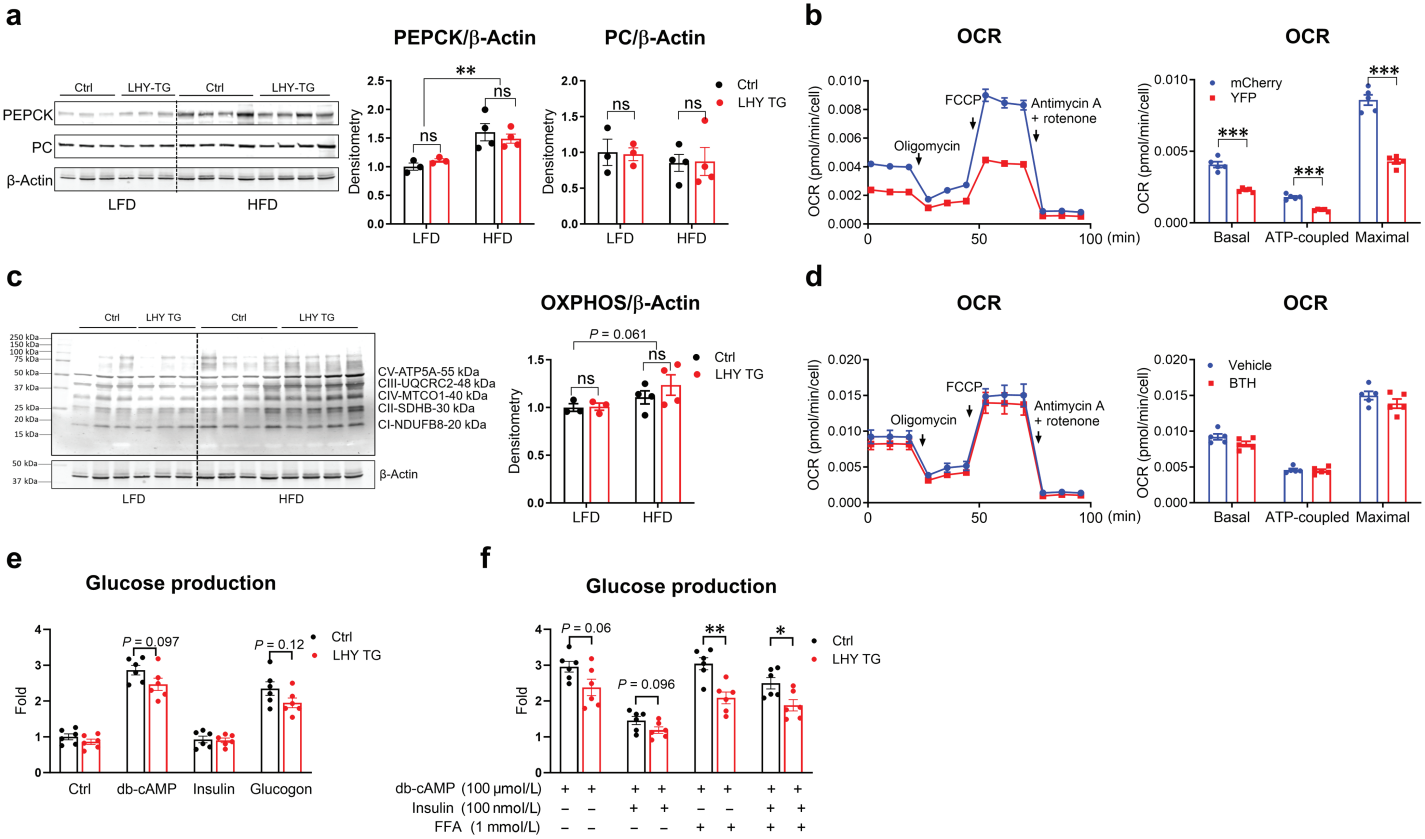
*Hyal1* overexpression reduces cellular ATP production-coupled respiration. (a) Western blot analysis of PEPCK and PC in liver lysates of control and LHY TG mice on LFD or HFD. Densitometry quantifications are shown on the right. (b) OCR measurement in AML12 cells stably overexpressing YFP or HYAL1 after serial injections of oligomycin, FCCP, and antimycin A + rotenone. Quantifications of basal OCR (before oligomycin injection), ATP-coupled OCR (difference between results before and after oligomycin injection), and maximal OCR (after FCCP injection) values are shown on the right. (c) Western blot analysis of mitochondrial OXPHOS proteins in liver lysates of control or LHY TG mice on LFD or HFD. Densitometry quantifications are shown on the right. (d) OCR measurement in HepG2 cells treated with BTH for 1 h followed by serial injections of oligomycin, FCCP, and antimycin A + rotenone. Quantifications of basal, ATP-coupled, and maximal OCR values are shown on the right. (e and f) Glucose production. Glucose production was assessed in primary mouse hepatocytes incubated for 8 h with db-cAMP (100 μmol/L), insulin (100 nmol/L), or glucagon (100 nmol/L), either in the presence (e) or absence (f) of FFAs (1 mmol/L). For panels (a) and (c), two-way ANOVA followed by *post-hoc* Sidak multiple comparison tests was performed. *n* = 3 and 4 mice for LFD and HFD groups, respectively. For panels (b) and (d), two-tailed *t*-tests were performed between treatment groups, *n* = 5 wells per group. β-Actin blots in panels (a) and (c) are the same. For panels (e) and (f), two-tailed *t*-tests were performed between treatment groups. *n* = 6 wells per group. All results are shown as mean ± SEM. ns, not significant. ^*^P < 0.05; ^**^P < 0.01; ^***^P < 0.001.

Gluconeogenesis is governed by key enzymes such as pyruvate carboxylase (PC) and phosphoenolpyruvate carboxykinase (PEPCK) [17]. HFD significantly increased PEPCK but not PC levels in the liver (Fig. 6a). However, protein abundance for both enzymes was not affected by *Hyal1* overexpression in the liver (Fig. 6a). Because gluconeogenesis is an energy-consuming process, we performed cellular energy measurements to understand how *Hyal1* overexpression affects cellular respiration. ATP-coupled oxygen consumption rate (OCR), an indicator of ATP generation, was significantly reduced in the *Hyal1* overexpressing AML12 cells (Fig. 6b); a similar pattern was also observed in HepG2 cells (Supplementary Fig. S6). The change in ATP generation is unlikely to be a result of changes in mitochondrial oxidative phosphorylation system (OXPHOS) protein complexes (Fig. 6c). By contrast, digestion of extracellular HA by bovine testis HYAL (BTH) did not affect the OCR values (Fig. 6d). All these data suggest that hepatocyte *Hyal1* remodels mitochondrial energetics, which may be responsible for reduced gluconeogenesis.

To evaluate the interactions between HYAL1-mediated HA degradation, metabolic hormones, and HFD-induced insulin resistance in glucose production, we isolated primary hepatocytes from control and LHY TG mice and measured glucose production under different conditions. First, hepatocytes isolated from overnight-fasted mice exhibited increased glucose output only in response to glucagon and dibutyryl-cAMP (db-cAMP), a membrane-permeable analog of cAMP, while HYAL1 showed a trend towards suppressing gluconeogenesis in both conditions. Notably, under low basal gluconeogenesis, insulin did not exert any additional suppression of glucose production (Fig. 6e). In the subsequent experiment, we included db-cAMP in all treatment conditions to mimic glucagon action in the fasting state. Then, we treated either isolated hepatocytes in the vehicle or free fatty acids (FFAs) for 16 h to induce insulin resistance and showed that the suppression of glucose output by insulin was significantly blunted by FFA treatment, but *Hyal1* expression still exerted additional inhibition of glucose output beyond the effect of insulin (Fig. 6f).

### HYAL1-mediated HA degradation reduces O-GlcNAcylation of ATP synthase subunits and reduces the enzymatic activity of ATP synthase

**Figure 7:**
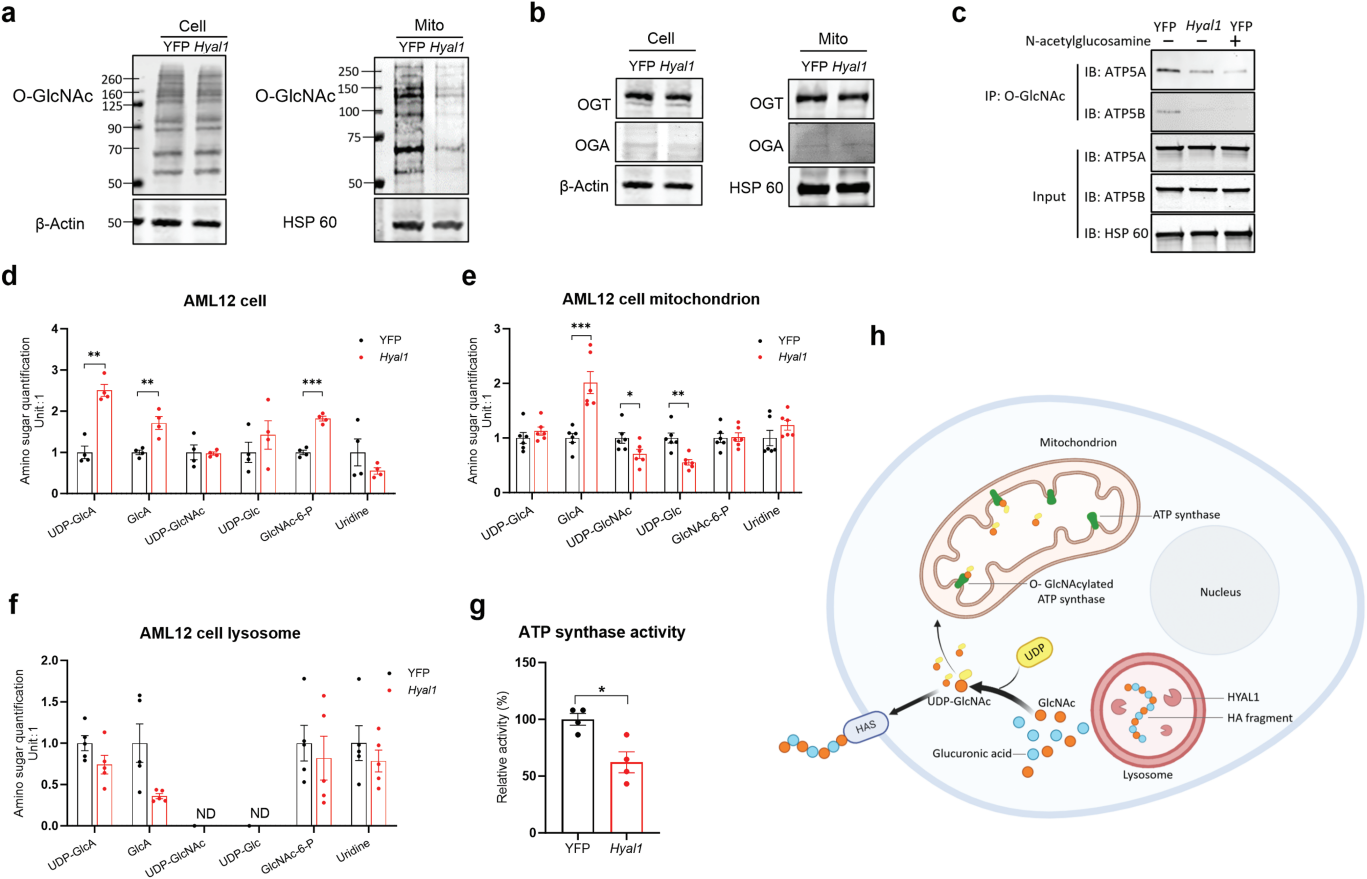
*Hyal1* overexpression reduces O-GlcNAcylation of ATP synthase subunits and reduces the enzymatic activity of ATP synthase. (a) O-GlcNAc levels in AML12 cells or their mitochondria. AML12 cells were stably transfected with YFP or *Hyal1*. (b) Protein levels of OGT and OGA in transfected AML12 cells and their mitochondria. Panel (a) used the same loading control as in panel (b). (c) Immunoprecipitation (IP) of O-GlcNAced mitochondrial protein with the RL2 antibody in mitochondria isolated from AML12 cells stably overexpressing YFP or *Hyal1*, followed by western blotting of ATP5A and ATP5B. GlcNAc preincubation before IP was used to show the specificity of the indicated detection antibody. Experiments (a), (b), and (c) were repeated at least one additional time. (d) Amino sugar quantification in transfected AML12 cells analyzed by mass-spectrometry. *n* = 4. (e) Amino sugar quantification in mitochondria of transfected AML12 cells. *n* = 5. (f) Lysosomes of transfected AML12 cells. *n* = 5. Abbreviations: UDP-GlcA: UDP glucuronic acid; GlcA: glucoronic acid; UDP-GlcNAc: UDP-N-acetylglucosamine; UDP-Glc: UDP-glucose; GlcNAc-6-P: N-acetylglucosamine-6-phosphate; GlcN: glucosamine; GlcN-6-P: glucosamine-6-phosphate. (g) Mitochondrial ATP synthase activity in AML12 cells stably overexpressing YFP or *Hyal1*. For panels (d)–(g), two-tailed *t*-tests were performed between treatment groups. (h) Schematic diagram of the mechanism by which HYAL1-mediated HA degradation regulates mitochondrial O-GlcNAcylation in postprandial hepatocytes through the redistribution of the HA catabolism metabolite GlcNAc and UDP-GlcNAc, which are involved in both HA synthesis and O-GlcNAcylation pathways. All results are shown as mean ± SEM. ^*^P < 0.05; ^**^P < 0.01; ^***^P < 0.001.

How does HYAL1-mediated HA degradation affect ATP production? HA degradation releases GlcNAc from the lysosomes. GlcNAc is quickly phosphorylated to reform UDP-GlcNAc via enzymatic reactions, which can then be reused for HA synthesis, O-GlcNAcylation, or other biological pathways. In AML12 cells stably expressing *Hyal1*, we found that overall cellular O-GlcNAcylation remained unchanged; however, mitochondrial O-GlcNAcylation was reduced (Fig. 7a). This was independent of the levels of O-GlcNAc transferase (OGT) and O-GlcNAcase (OGA), two key enzymes responsible for the dynamic process of O-GlcNAcylation (Fig. 7b). According to the literature, more than 10 proteins in mitochondria can be O-GlcNAcylated [20]. Among them, mitochondrial ATP synthase subunits 5A and 5B are essential components of the ATP synthase complex and play a crucial role in ATP synthesis during cellular respiration [21]. Immunoprecipitation (IP) of mitochondrial O-GlcNAc proteins using the RL2 anti-O-GlcNAcylated protein antibody, followed by detection of specific proteins, showed that the O-GlcNAcylation levels of ATP synthase subunits alpha and beta (ATP5A and ATP5B) were significantly reduced in *Hyal1* expressing cells, while the protein abundance was not affected by *Hyal1* expression (Fig. 7c).

To understand how mitochondrial protein O-GlcNAcylation was reduced independent of changes in OGT and OGA abundances, metabolites in the amino sugar pathway were quantified in whole AML12 cells, isolated lysosomes, and isolated mitochondria. HA will completely break down into GlcA and GlcNAc. GlcA and its derivative UDP-GlcA were significantly increased in *Hyal1-*overexpressing AML12 cells (Fig. 7d). The other product, GlcNAc, was not detected, probably due to quick phosphorylation to GlcNAc-6-P by GlcNAc kinase. Consistently with this, we observed elevated GlcNAc-6-P levels in *Hyal1-*overexpressing AML12 cells (Fig. 7d). Despite a similar increase of GlcA in mitochondria, GlcNAc-6-P was not different, and UDP-GlcNAc, the substrate of OGT and O-GlyNAcylation, decreased in the mitochondria (Fig. 7e). None of those metabolites were different in isolated lysosomes (Fig. 7f), indicating the dynamic nature of lysosomes and quick trafficking of those metabolites. In addition, due to numerous phosphatases and nucleotidases [22], UDP-GlcNAc and UDP-Glc were not detected in the lysosome (Fig. 7f).

The decrease in mitochondrial O-GlcNAcylation corresponded with a reduction in ATP synthase activity (Fig. 7g), aligning with the results previously reported in HeLa cells [23]. All these data point to a repartitioning of the subcellular UDP-GlcNAc that mediates mitochondrial protein O-GlcNAcylation and mitochondrial function, subsequently regulating mitochondrial ATP production, eventually contributing to the suppression of gluconeogenesis (Fig. 7h).

## Discussion

This paper studied the role of postprandial surges of circulating HA in hepatic gluconeogenesis. Starting by observing impaired glucose tolerance and elevated gluconeogenesis in *Hyal1* KO mice, we pinned down the liver as the primary organ contributing to this metabolic phenotype on the basis of high expression of *Hyal1* in the liver, hepatic HA accumulation in *Hyal1* KO mice [12], and the fact that the liver is the primary organ for gluconeogenesis [17]. Then, with hepatic *Hyal1* overexpression mice, primary hepatocytes, and hepatocyte cell lines, we identified the role of HYAL1-mediated HA degradation in suppressing gluconeogenesis.

Hepatic metabolism is actively regulated by the expression of enzymes catalyzing key reactions in these metabolic pathways. However, *Hyal1* deletion did not affect gene expression overall. So, how does HYAL1 regulate gluconeogenesis? In fact, the HA catabolism end product GlcNAc is released from the lysosome and converted into GlcNAc-6-P and UDP-GlcNAc to regulate cellular signaling and metabolism. This is done through subcellular compartmentalization of the UDP-GlcNAc and reduced allocation of UDP-GlcNAc to mitochondria to specifically reduced O-GlcNAcylation levels of ATP5A and ATP5B, resulting in reduced mitochondrial ATP synthase activity and reduced ATP production, which are expected to affect gluconeogenesis.

Compartmentalization of the cells and heterogenous subcellular distribution of metabolites can be achieved with membrane organelles, by phase separation, or by specialized subcellular location of key enzymes, which work together with biophysical limits of diffusion to form a static-appearing concentration gradient within the cell. Our study showed a significant increase in *Has* expression in livers overexpressing *Hyal1* (Fig. 3l) or after refeeding (Supplementary Fig. S1b), suggesting a compensatory HA synthesis at the plasma membrane under those scenarios [13]. This compensatory HA synthesis may drive a flux of cellular UDP-GlcNAc towards the plasma membrane, leading to reduced allocation to mitochondria.

It is essential to point out that mitochondrial protein O-GlcNacylation is a debated topic, especially regarding whether this modification occurs in the mitochondria or before these proteins are imported into the mitochondria, because OGT is localized predominantly in the nucleus and cytoplasm [24, 25], with only a small fraction in the mitochondria. It is possible that mitochondrial OXPHOS complexes (e.g. ATP5A/ATP5B) are synthesized on the endoplasmic reticulum (ER) and undergo O-GlcNacylation before they are imported into the mitochondria. Further, it remains possible that there is a UDP-GlcNAc gradient that is low around the ER, leading to reduced O-GlcNacylation of mitochondrial proteins. It is also likely that both processes occur in parallel and contribute collectively to this postprandial metabolic regulation.

We also observed that the effect of HYAL1 on gluconeogenesis occurred primarily in mice subjected to HFD and that HYAL1 still inhibited glucose production on top of insulin in FFAs-induced insulin-resistant primary hepatocytes. The differential impact can be attributed to the metabolic stress imposed by HFD and exposure to FFAs. In the classical view, postprandial suppression of gluconeogenesis is mediated by the surge of insulin and decrease of glucagon after a meal: insulin suppresses gluconeogenesis directly in the liver and indirectly via other peripheral tissues, such as adipose tissue, by suppressing the release of FFAs and glycerol. With HFD-induced obesity, the fatty liver becomes insulin-resistant and inefficient in these processes, failing to suppress gluconeogenesis and contributing to hyperglycemia. In contrast, HYAL1-HA regulates gluconeogenesis differently: HAYL1 is constantly expressed, and its effect is enhanced by a surge of postprandial blood HA levels, which have the same magnitude for HFD and LFD. At the molecular level, the HYAL1-HA pathway affects ATP production to regulate the same gluconeogenesis pathway that insulin and glucagon regulate. Because the HYAL1-HA pathway is not affected by insulin resistance, it manifests a more significant effect in fatty livers from HFD-treated mice. In addition, ATP levels are lower in those livers than in lean and insulin-sensitive mice due to mitochondrial dysfunction [26, 27] and increased uncoupling [28], making the livers of HFD mice more vulnerable to decreased ATP and thus causing a more pronounced suppression of gluconeogenesis. All these data support a model that HYAL1-HA, glucagon, and insulin convene on the same metabolic pathway in regulating glucose usage and gluconeogenesis.

Physiologically, gluconeogenesis generates glucose from non-carbohydrate precursors to support critical organs like the brain and red blood cells during fasting and other conditions when dietary glucose is unavailable. It is tightly regulated, and failure to suppress it after a meal leads to high postprandial glucose levels in type 2 diabetic patients [29]. In parallel to insulin action, the direct release of HA from the gastrointestinal tract, which then travels through the portal vein to the liver to be digested by hepatocyte HYAL1 to inhibit gluconeogenesis, offers another level of regulation of postprandial gluconeogenesis, which is especially important in the livers with insulin resistance. A lack of such regulation would lead to increased gluconeogenesis, explaining why the anti-diarrhea medicine loperamide unwantedly increases blood glucose levels in human subjects [30].

In conclusion, we found that HYAL1*-*mediated HA degradation inhibits gluconeogenesis in parallel to classical insulin action in the liver of HFD-fed mice—a mechanism that could be potentially enhanced to inhibit gluconeogenesis in insulin-resistant patients.

### Limitations of the study

While our findings underscore the importance of HYAL1-mediated HA degradation in suppressing gluconeogenesis, the relative contribution of this pathway compared to classical insulin signaling remains unclear. Moreover, our study primarily assessed gluconeogenesis from precursors such as pyruvate, using glucose as the output measure, and did not evaluate glycerol-derived gluconeogenesis, which may be differentially influenced by HYAL1-HA metabolism. Future studies employing more advanced stable isotope tracing techniques to quantify the interplay between insulin, HA, and alternative gluconeogenic substrates could offer a more comprehensive understanding of postprandial glucose regulation.

## Materials and methods

### Animals

*Hyal1* ^tm1Stn/Mmcd^ (*Hyal1* KO) mice were generated previously [31] and obtained from Mutant Mouse Regional Resource Centers (MMRRC). The mice were backcrossed to a C57Bl/6 genetic background for nine generations before the experiments [32]. TRE-*Hyal1* was generated by subcloning mouse cDNA into a pTRE vector (Clontech) with a rabbit β-globin 3'-UTR. Linearized DNA was injected into an oocyte, and the founders were identified through PCR reactions. The founders were then crossed with Adipoq-rtTA mice (Jax #033448) [33] or albumin-*Cre* transgenic (Jax #003574) [34] and Rosa26-loxP-STOP-loxP-*rtTA* transgenic (Jax #005572) [35] double transgenic mice to select a founder mouse that could achieve tissue-specific transgene induction after doxycycline treatment and had no leakage of transgene expression into other tissues for the subsequent experiments. All animals were kept on a 12-h light/12-h dark cycle in a temperature-controlled environment. Mice were free to access water and were fed one of the following: a standard chow diet (LFD), a 60% HFD (BioServ, S1850), or a 60% HFD containing 200 mg/kg doxycycline (BioServ, S6223). Mice were genotyped by Transnetyx. The *Hyal1* KO study was performed at the University of Namur; the rest of the animal studies were performed at the Baylor College of Medicine. Animal care and experimental protocols were approved by the Institutional Animal Care and Use Committee of the University of Namur and the Baylor College of Medicine.

### HA extraction and quantification

The procedure was performed as previously described [16], and the extracted HA was quantified with an ELISA kit (R&D systems, DHYAL0), using a sample digested with BTH overnight as the negative control. The tissue HA content was normalized to the wet weight used for extraction.

### Histological analysis

After the mice were euthanized, the tissue was excised immediately, fixed overnight in 10% PBS-buffered formalin, and stored in 50% ethanol. Tissues were sectioned (5 µm), rehydrated, and stained with hematoxylin and eosin (H&E) at the Pathology Core of the Baylor College of Medicine. Microscopic images were taken on a ZEISS Axioscan scanner.

### Western blotting

Protein extractions were performed as previously described [36]. Protein abundance was detected using primary antibodies against GLUT1 (Novus Biologicals, NB110-39113SS, 1:1,000 dilution), GLUT2 (Novus Biologicals, NBP2-22218SS, 1:500 dilution), PEPCK/PCK2 (Abclonal, A4466, 1:1,000 dilution), PC (Abclonal, A8980, 1:1,000 dilution), O-‍GlcNAc (Novus Biologicals, NB300-524SS, 1:1,000 dilution), total OXPHOS (Abcam, ab110413, 1:1,000 dilution), ATP5B (Novusbio, NBP3-15354, 1:1,000 dilution), HSP60 (Novusbio, NBP1-77397SS, 1:1,000 dilution), or β-Actin (Cell Signaling Technology, 3700S, 1:1,000 dilution). Secondary antibodies used included goat anti-mouse, Alexa Fluor Plus 800 (Invitrogen, A32730), and goat anti-rabbit, Alexa Fluor Plus 680 (Invitrogen, A32734), both at 1:10,000 dilutions. Antibody-decorated membranes were then visualized on a Li-Cor Odyssey infrared scanner, and the scanned data were analyzed using Odyssey version 3.0 software.

### Metabolic measurements

GTTs were performed as described previously [16]. In brief, the mice were fasted for 4−6 h, after which glucose solution (10 µL/g body weight) was administered orally or by intraperitoneal injection; the final dose of glucose was 1.25 g/kg body weight for the mice on LFD and 0.75 g/kg body weight for the mice on HFD. Blood glucose levels before and 15, 30, 60, and 120 min after the glucose injection were measured using a glucometer. Serum insulin levels were measured using the ALPCO Mouse Insulin ELISA Jumbo kit (cat. number 80-INSMS-E10). PTTs were performed on the overnight fasted mice with or without cisapride (Cayman Chemical Company, 21657) treatment. Basal blood glucose levels were measured in the morning. Cisapride was dissolved in DMSO and then diluted in PBS to 0.05 mg/mL. It was administered by oral gavage at 10 μL/g body weight (0.5 mg/kg body weight) or vehicle (0.5% DMSO in PBS) to mice 1 h before pyruvate injection. Sodium pyruvate was dissolved in water and injected intraperitoneally at a final dose of 1 g/kg body weight. Blood glucose levels before and 15, 30, 60, and 120 min after the glucose injection were measured using a glucometer. Insulin tolerance tests (ITTs) were performed on mice fasted for 4−6 h. Human insulin (Sigma (Roche), 11376497001) was dissolved in saline and injected peritoneally; the final dose was 1 IU/kg body weight. Blood glucose levels before and 15, 30, 60, and 120 min after the glucose injection were measured using a glucometer. Triglyceride clearance tests were performed as described previously [37]. In brief, the mice were fasted overnight, and then 20% intralipid (Sigma, I141-100 mL) was orally gavaged at 15 µL/g body weight. Blood glucose levels before and 1.5, 3, and 6 h after intralipid administration were taken from the tail, and serum triglyceride levels were measured using Infinity triglyceride reagents (Thermo Fisher, TR22421).

### Serum lipid measurements

Serum triglycerides were measured using Infinity reagents. Serum NEFA was measured by NEFA-HR assay (Wako, C1057). Total cholesterol and HDL cholesterol were measured using an AF HDL and low-density lipoprotein/very low-density lipoprotein (LDL/VLDL) assay kit (Sigma-Aldrich, MAK331). The measurements were carried out following manufacturer-provided protocols.

### Metabolomics

The metabolites were extracted from the mouse liver tissues [38]. The TCA cycle and glycolysis metabolites and their intermediates were separated using a Luna 3 µm NH2 (100 A°) HPLC column, and 5 mmol/L ammonium acetate in water (pH 9.9) and acetonitrile were used as mobile phase solvents. The metabolites were separated through the Agilent 1290 Infinity HPLC system, and the data were acquired using Agilent 6495B Triple Quadrupole mass spectrometry via multiple reaction monitoring (MRM) in negative ionization mode [38, 39]. These data were analyzed using Agilent Mass Hunter quantitation software. The data were normalized with a spiked isotopically labeled internal standard, and relative abundance was plotted.

### Quantitative real-time PCR (qRT-PCR)

Using a reverse transcription kit (Bio-Rad), 1 µg RNA was used to transcribe cDNA. qRT-PCR primers were obtained from the Harvard PrimerBank [40]; these are listed in Supplementary Table S3. The messenger RNA levels were calculated using the comparative threshold cycle method, normalized to gene *Rps16*.

### RNA-seq

RNA was isolated from frozen tissues by homogenization in Trizol reagent (Invitrogen, 15596018) as previously described [41]. RNA concentrations were quantified using a NanoDrop Spectrophotometer, and sample integrity was verified using an Agilent 2100 Bioanalyzer (Agilent Technologies). Only samples with RNA integrity number (RIN) values above 8.0 were used for experiments. cDNA libraries were prepared using an Illumina TruSeq RNA sample prep kit. The average size of the library cDNAs was 150 bp (excluding adapters). The integrity and quality of the cDNA libraries were assessed using an Agilent 2100 Bioanalyzer and an ABI StepOne Plus real-time PCR system. RNA-seq was performed by the Novogene. Raw read sequencing quality and adapter contamination were assessed using FastQC v0.11.9. The overall quality was determined to be satisfactory, and raw reads were aligned to the mouse genome index using STAR v2.7.9a. The STAR genome index was created using raw FASTA and annotation files downloaded from the GENCODE portal for mouse genome, build GRCm38, release 23. Alignments were saved in binary format (BAM). Summaries of raw read quality and alignment quality were generated using MultiQC v1.12 [42]. Sample-specific gene expression values were computed as the number of reads aligned per gene using STAR–quantMode GeneCounts. Raw counts were normalized, and genes with an average read count of < 50 across all samples were considered unexpressed and were excluded from the differential analysis. The analysis for differential gene expression was carried out using DESeq2 [43]. A false discovery rate (FDR) cut-off of 0.05 and a fold change cut-off of 20% (−0.263 ≤ log_2_(Fold change) ≥+0.263) were imposed to identify significantly differentially expressed genes. Genes identified in different contrasts were then overlapped to determine the directionality of differential gene expression between the contrasts. The accession number for the RNA-seq data is GEO: GSE220752.

### IP

AML12 cells were transfected with plasmids encoding HYAL1 or YFP. Stable transgenic cell lines were established by hygromycin (Thermo Fisher, 10687010) selection.

Direct IP with O-GlcNAc antibody or ATP5B antibody was carried out as follows. O-GlcNAc antibody RL2 (Novusbio, NB300-524) or ATP5B antibody (Novusbio, NBP3-15354) was conjugated to NHS-activated magnetic beads (Thermo Scientific, 88828). The antibody-coupled beads were then incubated with AML12 YPF/HYAL1 mitochondrial protein for 2 h at room temperature, with or without 6.7 mmol N-Acetylglucosamine (Sigma-Aldrich, PHR1432-1G). After incubation, the precipitates were washed with cell lysis buffer to remove non-specifically bound proteins. The bound proteins were then eluted and analyzed with western blot analysis.

Classic IP with ATP5B antibody was carried out as follows. Mitochondrial protein lysates from YFP and *Hyal1*-overexpressing cells were incubated with ATP5B antibody for 2 h at room temperature. The immune complexes were captured using protein A/G magnetic beads. Western blot analysis was performed to compare the O-GlcNAcylation levels between the YFP and *Hyal1* overexpressing mitochondrial protein samples, using RL2 or 9D1.E4 antibodies (Invitrogen, MA1-039).

### ATP synthase activity assay

ATP synthase activity was measured using the ATP Synthase Enzyme Activity Microplate Assay Kit (ab109714) following the manufacturer’s protocol. Mitochondria were isolated from AML12 cell lines stably expressing mCherry or HYAL1 as previously described [44]. The isolated mitochondria were lysed and diluted to the desired concentration in 4-fold volume of a buffer solution. Then 1/10 volume of the provided detergent was added, mixed thoroughly, and incubated on ice for 30 min. The mixture was centrifuged, and the supernatant was collected for further analysis. For the assay, 50 µL of the sample was added to each well of a 96-well microplate coated with a specific monoclonal antibody, and incubated at room temperature for 3 h to ensure complete enzyme−antibody binding. The plate was then washed twice with buffer solution to remove unbound material. Subsequently, 40 μL of LIPID MIX was added to each well, and the reaction was incubated at room temperature for 45 min. Following this, 200 µL of the detection reagent was added to each well. Absorbance at 340 nm was measured at 30°C using a kinetic program on a microplate reader (BMG Labtech FLUOstar Omega Microplate Reader) over 60−120 min. ATP synthase activity was determined by evaluating the rate of decrease in absorbance at 340 nm over time.

### Measurement of cellular energetics

The experiment was carried out on the Agilent Seahorse XFe24 instrument in accordance with the Agilent Seahorse protocol. In detail, HepG2 cells were transfected with indicated constructs (1 µg/well in a 12-well plate using PolyJet DNA transfection reagent (SignaGen)) and seeded into an Agilent Seahorse XF24 cell culture microplate at a density of 60,000 cells per well (Wells A1, B4, C3, and D6 were used as the background, without cells) on the second day. In parallel, an Agilent Seahorse XFe24 extracellular flux assay plate with 1 mL Seahorse XF calibrant solution was equilibrated overnight at 37°C. The next day, a Mito stress test was performed using Palmitate-BSA as described, using an *in vitro* Seahorse XF Cell Mito Stress Test Kit, with the sequential injection of the following compounds: Port A: oligomycin, with a final concentration of 2 µmol/L; Port B: carbonyl cyanide-p-(trifluoromethoxy)phenylhydrazone (FCCP), with a final concentration of 4 µmol/L; Port C: rotenone, with a final concentration of 1 µmol/L, and antimycin A, with a final concentration of 4 µmol/L. All final concentrations were in the culture medium after injection.

### Hepatocyte isolation

Primary hepatocytes were isolated from control and LHY TG mice fasted for 24 h to deplete liver glycogen, following a previously described protocol [45]. In brief, 8- to 10-week-old mice were anesthetized with 100 mg/kg ketamine (Covetrus) and 10 mg/kg xylazine (Rompun), and then perfused at 3 mL/min for 15 min (37°C) with a washing buffer (Hanks balanced salt solution (HBSS) without Ca²⁺/Mg²⁺/phenol red (ThermoFisher, 88284), 25 mmol/L HEPES, and 0.5 mmol/L EDTA, pH 7.4) to remove blood. The liver was then digested (2 mL/min, 10 min, 37°C) with HBSS containing Ca²⁺/Mg²⁺/phenol red (ThermoFisher, 14025076), 25 mmol/L HEPES (Boston Bioproducts, BBH-74), and 25 μg/mL Liberase (Sigma Aldrich, 5401119001). After excision, the liver was placed on ice, ruptured to release cells, filtered (70 μm), and centrifuged (50 *g*, 2 min, 4°C). Hepatocytes were further purified by Percoll (VWR, 89428-524) gradient centrifugation (200 *g*, 10 min, 4°C), and then resuspended in Williams’ Medium E (Thermo Fisher, 32551020) with 10% fetal bovine serum (Thermo Fisher, 16140071), penicillin (100 UI/mL), and streptomycin (100 μg/mL) (Sigma Aldrich, P4333-100mL). Only isolates with ≥ 90% viability were used. Cells (1.25 × 10⁵/well, 24-well plates) were seeded on collagen-coated plates, incubated for 4 h, and then switched to a fresh medium for further experimentation.

### Preparation of 1-mmol/L palmitic acid (PA):oleic acid (OA) (1:2) FFA-BSA complex solution

A 1-mmol/L PA:OA (1:2) FFA-BSA complex was prepared by mixing PA and OA bound to BSA. Specifically, 660 μL of BSA-palmitate complex (5 mmol/L PA and 0.8 mmol/L BSA; Cayman Chemical, 29558) and 2.23 mL of OA-albumin solution (3.0 mmol/L OA and 1.5 mmol/L BSA; Sigma-Aldrich, 03008) were combined. The mixture was diluted to a final volume of 10 mL with fatty acid-free DMEM (approximately 7.11 mL of buffer). The solution was gently heated at 37°C and vortexed to ensure complete mixing. The final solution contained 1 mmol/L total fatty acids (PA:OA = 1:2) and approximately 0.36 mmol/L BSA. Subsequently, the solution was filtered through a 0.22-μm membrane for sterilization. The prepared medium was immediately used to replace the cell culture medium. Equal amounts of BSA were added to the control cells in each experiment.

### Assessment of glucose production in primary hepatocytes

Glucose production in primary hepatocytes was evaluated as previously described [46]. In brief, cells were cultured in Williams’ Medium E supplemented with 10% fetal bovine serum, followed by three washes with warm PBS to remove residual glucose. Cells were then treated with 100 μmol/L db-cAMP (Sigma Aldrich, D0260-5mg), 100 nmol/L insulin, or 100 nmol/L glucagon (Genscript, RP10772) in glucose-free Dulbecco’s Modified Eagle’s Medium (Gibco, 11966025) containing gluconeogenic substrates (20 mmol/L sodium lactate (Sigma, 1614308) and 2 mmol/L sodium pyruvate (Sigma, 792500-100G)) and 200 μg/mL sodium hyaluronate (Lifecore Biomedical, HA200K-1, MW = 200 kDa). For the insulin resistance model, cells were pretreated with 1 mmol/L oleate/palmitate (2:1) mixture for 16 h, thoroughly washed with warm PBS, and subsequently subjected to the same stimulation protocol. After 6 h, the cell culture supernatants were collected to measure the glucose content using a Roche glucose assay kit (cat. no. 0716251) and normalized to total protein content. Total glucose production comprised contributions from both glycogenolysis and gluconeogenesis. Glycogenolysis-derived glucose production was measured in the absence of gluconeogenic substrates, while gluconeogenic glucose production was determined as the difference between total glucose output and glycogenolysis.

### Statistical analysis

Results are shown as mean ± standard error of the mean (SEM). For experiments with only two groups, Student’s *t*-test was used. For studies with three or more groups, one-way ANOVA was used; for experiments with several groups with a balanced distribution of two factors, a two-way ANOVA test was used. A Sidak test was used for *post-hoc* analysis of comparisons within subgroups. *P* values of < 0.05 were considered statistically significant. More details are provided in the figure legends.

## Supplementary Material

loaf016_suppl_Supplementary_Figures_S1-S6

loaf016_suppl_Supplementary_Table_S1

loaf016_suppl_Supplementary_Table_S2

loaf016_suppl_Supplementary_Table_S3

## Data Availability

The authors confirm that all the data supporting the findings of this study are available within the supplementary material and corresponding authors.
