# Supplementary material for: Hyaluronidase-1 mediates postprandial suppression of hepatic gluconeogenesis
Source: Life Metab. 2025 May 13;4(5):loaf016. doi: 10.1093/lifemeta/loaf016 (PMC13122362; doi:10.1093/lifemeta/loaf016)
Supplement: loaf016_suppl_Supplementary_Figures_S1-S6 [file loaf016_suppl_supplementary_figures_s1-s6.pdf]

Hyaluronidase-1 mediates postprandial suppression of hepatic gluconeogenesis

Xi Chen[1], Sophie Dogné[2], Yanru Deng[1], Huiqiao Li[1], Jieyi Meng[1], Charlise Giang[1], Jan-Bernd Funcke[3], Leon G. Straub[4], Michelle Dias[1, 5], Sundararajah Thevananther[6], Qiang Tong[1], Abu Hena Mostafa Kamal[7], Chandra Shekar R. Ambati[8], Yu'e Liu[9], Nagireddy Putluri[7], Xia Gao[1], Miao-Hsueh Chen[1], Dongyin Guan[10], Hari Krishna Yalamanchili[1,5], Shangang Zhao[11], Nathalie Caron[2], Yi Zhu[1,*]

Table of Contents

Supplementary figures……………………………………………………………………….2

Supplementary Figures

Supplementary Figure S1

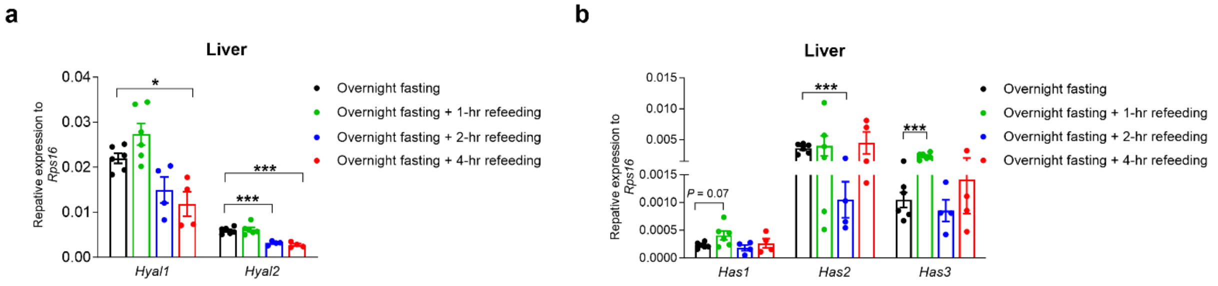

Supplementary Figure S1 Expression of (a) *Hyal* and (b) *Has* after overnight fasting and refeeding. *n* = 6 mice for overnight fasting and overnight fasting + 1-h refeeding groups; *n* = 4 mice for overnight fasting + 2-h refeeding and 4-h refeeding groups. All results are shown as mean ± standard error of the mean (SEM). $^{*}P < 0.05$; $^{***}P < 0.001$.

Supplementary Figure S2

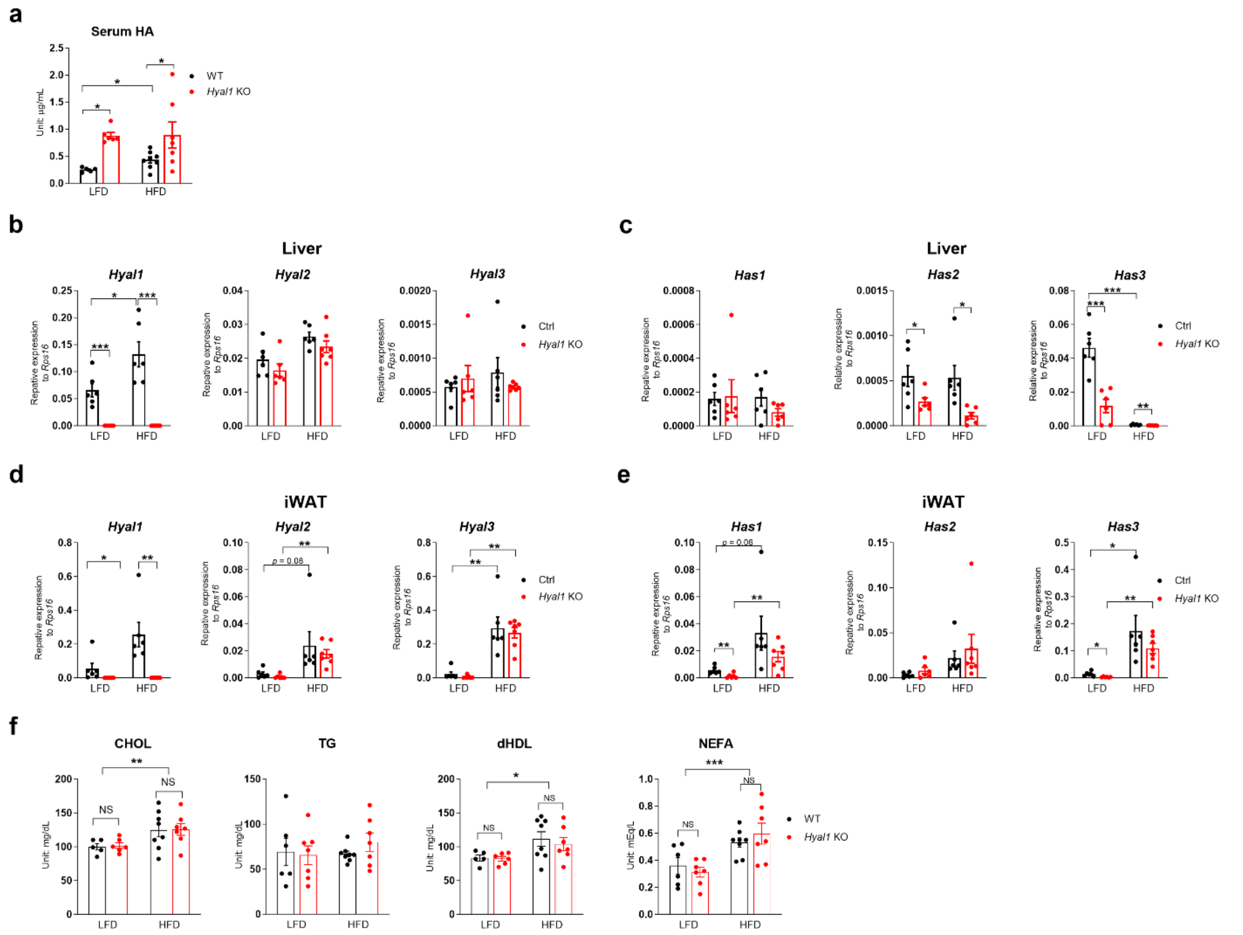

Supplementary Figure S2 Expression of HA metabolic genes in *Hyal1* KO liver and adipose tissue. (a) Serum HA levels for WT and *Hyal1* KO mice kept on LFD and HFD for 11 weeks. *n* = 5 mice for WT and *Hyal1* KO groups on LFD; *n* = 8 mice for WT and *Hyal1* KO groups on HFD. (b) Expression of *Hyal1*, *Hyal2*, and *Hyal3* in the livers of WT and *Hyal1* KO mice kept on LFD or HFD for 11 weeks. (c) Expression of *Has1*, *Has2*, and *Has3* in the livers of WT and *Hyal1* KO mice kept on LFD or HFD for 11 weeks. (d) Expression of *Hyal1*, *Hyal2*, and *Hyal3* in inguinal white adipose tissue (iWAT) from WT and *Hyal1* KO mice kept on LFD or HFD for 11 weeks. (e) Expression of *Has1*, *Has2*, and *Has3* in iWAT from WT and *Hyal1* KO mice kept on LFD or HFD for 11 weeks. In panels (b)–(e), *n* = 7 mice for the group of *Hyal1* KO mice on HFD, *n* = 6 mice for the other groups. Two-way ANOVA tests followed by *post-hoc* Sidak multiple comparison tests were used for panels (a)–(e). (f) Serum lipid profile of male WT and *Hyal1* KO mice kept on LFD and HFD for 11 weeks. CHOL: cholesterol; dHDL, direct high-density lipoprotein; NEFA: non-esterified free fatty acid; TG, triglyceride. *n* = 6 for control mice on LFD; *n* = 7 for *Hyal1* KO mice on LFD; *n* = 8 for control mice on HFD; *n* = 7 for *Hyal1* KO mice on HFD. Two-way ANOVA tests were used, dietary treatment effects were labeled, and Y values from different genotypes (WT and *Hyal1* KO) did not differ. All results are shown as mean ± SEM. NS: not significant. $^{*}P < 0.05$; $^{**}P < 0.01$; $^{***}P < 0.001$.

Supplementary Figure S3

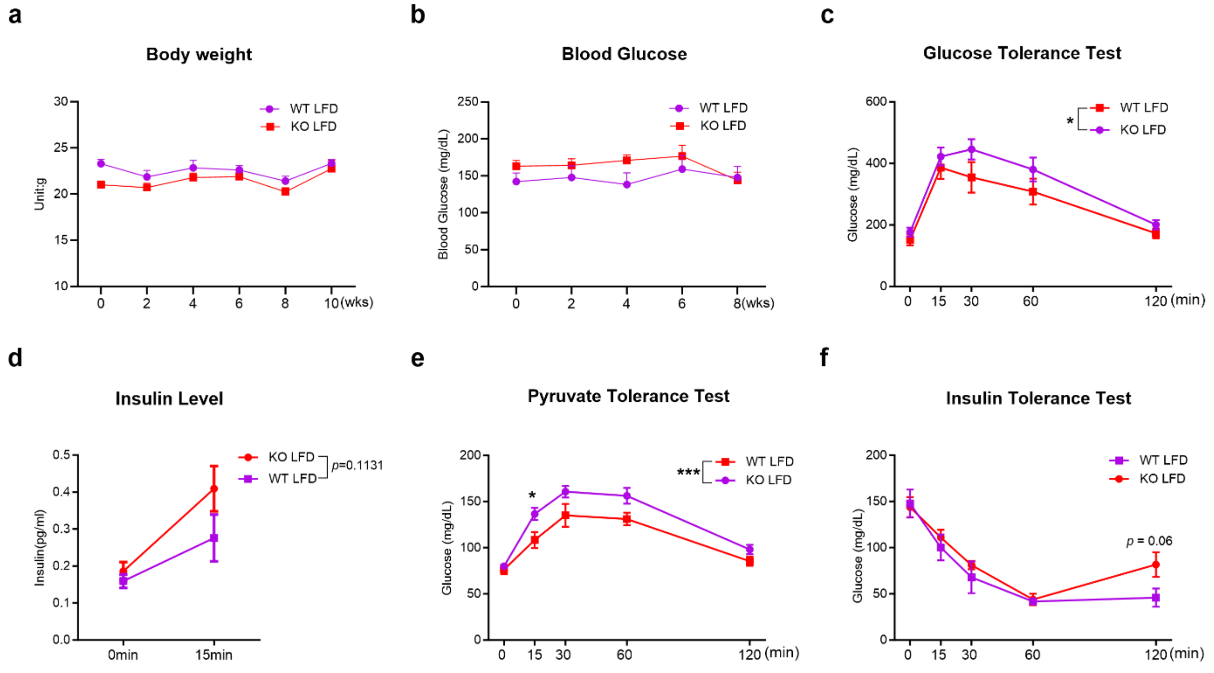

Supplementary Figure S3 *Hyal1* deficiency impairs glucose homeostasis. (a) Body weight. (b) Glycemia in random fed state. (c) OGTT. (d) Serum insulin levels during OGTT. (e) PTT. (f) ITT of female WT and *Hyal1* KO mice on LFD at 17–19 weeks old (matching the experimental age of male mice). n = 7 mice for WT group; n = 9 mice for *Hyal1* KO group. Two-way ANOVA tests followed by *post-hoc* Sidak multiple comparison tests were used between WT and *Hyal1* KO mice at different time points. Asterisk indicates statistical differences in Y values at indicated time points between WT and *Hyal1* KO mice for panels (a)–(f). All results are shown as mean ± SEM. $^{*}P < 0.05$; $^{**}P < 0.01$; $^{***}P < 0.001$.

Supplementary Figure S4

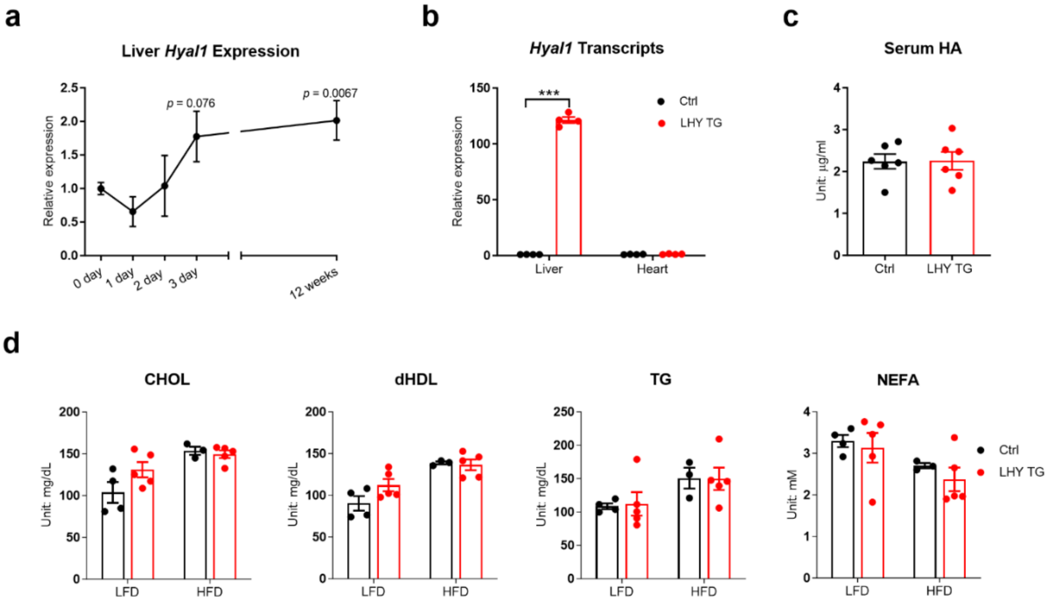

Supplementary Figure S4 (a) Hepatic *Hyal1* expression in C57BL/6J male WT mice treated with HFD. Serial *t*-tests were used to compare different time points to time = 0 min. *n* = 14, 10, 10, 9, and 12 mice for 0-day, 1-day, 2-day, 3-day, and 12-week groups, respectively. (b) *Hyal1* founder screening and hepatic overexpression of *Hyal1* after 5-day Dox200 LFD treatment. *n* = 4 mice for each group. (c) Serum HA concentration of control and LHY TG mice treated with Dox200 HFD for 8 weeks. *n* = 6 mice for each group. (d) Serum lipids in male control and LHY TG mice treated with Dox200 LFD or Dox200 HFD for 12 weeks. CHOL: cholesterol; dHDL, direct high-density lipoprotein; NEFA: non-esterified free fatty acid; TG: triglyceride. *n* = 3 and 5 for control and LHY TG mice, respectively, on LFD; *n* = 4 and 5 for control and LHY TG mice, respectively, on HFD. Student's *t*-tests were used for panels (b) and (c). Two-way ANOVA was performed for panel (d). All results are shown as mean ± SEM. $^{***}P < 0.001$.

Supplementary Figure S5

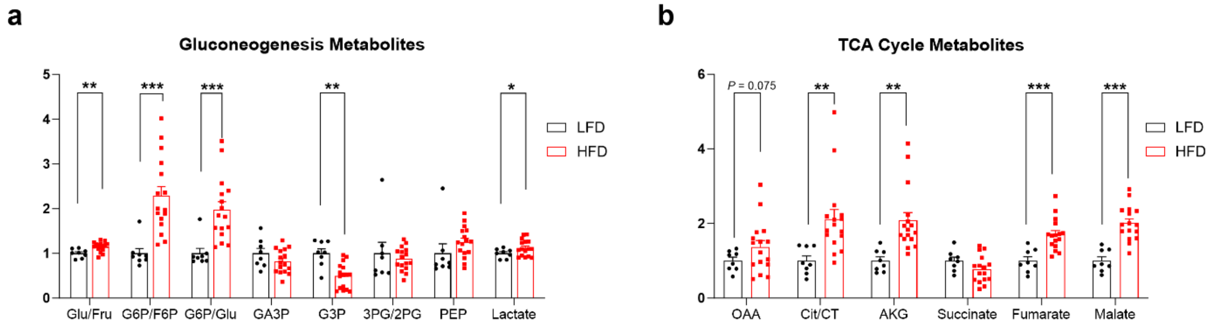

Supplementary Figure S5 Liver metabolites in the gluconeogenesis and TCA pathways after HFD treatment. (a) Metabolites in gluconeogenesis pathway in livers from LFD or HFD mice. (b) Metabolites in the TCA pathway in livers from LFD or HFD mice. Two-way ANOVA followed by *post-hoc* Sidak multiple comparison tests were performed. $n = 8$ mice for the LFD group and $n = 16$ mice for the HFD group. All results are shown as mean ± SEM. $^{*}P < 0.05$; $^{**}P < 0.01$; $^{***}P < 0.001$.

Supplementary Figure S6

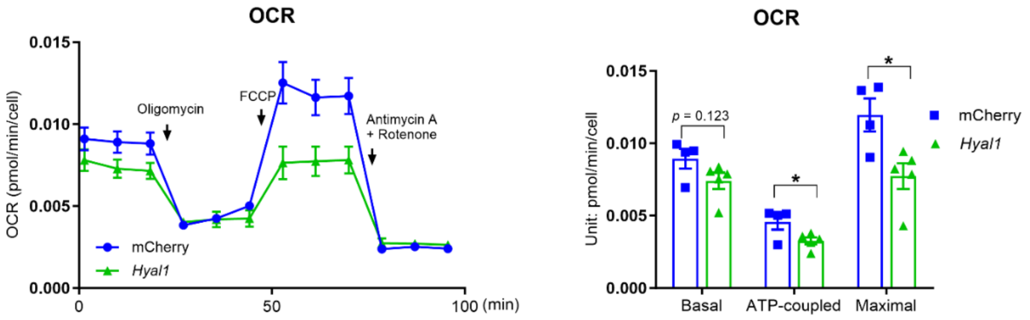

Supplementary Figure S6 Oxygen consumption rate (OCR) in HepG2 cells transiently overexpressing mCherry or *Hyal1* overnight after serial injections of oligomycin, carbonyl cyanide-*p*-(trifluoromethoxy)phenylhydrazone (FCCP), and antimycin A + rotenone. Quantifications of basal OCR (before oligomycin injection), ATP-coupled OCR (the difference before and after oligomycin injection), and maximal OCR (after FCCP injection) are shown on the right. Two-tailed *t*-tests were performed between treatment groups. $n = 5$ wells for *Hyal1* group; $n = 4$ for mCherry group. one sample was excluded from the mCherry group in panel (c) due to the sensor's failure in the well. All results are shown as mean ± SEM. $^{*}P < 0.05$; $^{**}P < 0.01$; $^{***}P < 0.001$.
